# Appendicitis in non-typhoidal salmonella bacteraemia

**DOI:** 10.1093/omcr/omy082

**Published:** 2018-10-22

**Authors:** Siow Yun Wong, Samuel Kang Lian Lee, Chaozer Er, Navin Kuthiah

**Affiliations:** 1 Ministry of Health Holdings, Singapore; 2 Department of General Medicine, Woodlands Health Campus, Singapore

## Abstract

Salmonella typhi and paratyphi infections can manifest as acute abdomen due to intestinal perforations, salpingitis and rarely appendicitis. Non-typhoidal salmonella infection that usually only causes self-limiting gastroenteritis, is rarely associated with appendicitis. We present the case of a 78-year-old gentleman with Salmonella bacteraemia complicated by acute appendicitis. He was treated conservatively due to multiple comorbidities. His condition improved after completion of 2 weeks of antibiotics guided by the blood and stool culture results. Appendicitis is a rare but important complication to consider in Salmonella bacteraemia. More research needs to be done with regards to the clinical course of Salmonella related appendicitis.

## INTRODUCTION

Bacteraemia is a serious extra-intestinal complication of Salmonella infection that is more common in elderly patients with chronic or immunosuppressing conditions [1]. Based on two studies, the incidence of non-typhoidal salmonella bacteraemia is only ~5–10% [2]. An association between Salmonella bacteraemia and acute appendicitis has previously been reported but is poorly understood, and difficult to differentiate from other conditions mimicking appendicitis such as mesenteric adenitis [3]. While management of acute appendicitis is primarily surgical and appendicitis can potentially be a surgical emergency, accurate diagnosis of appendicitis and prompt surgical management can be influenced especially in such cases where the patient is septic and appendicitis presents in an atypical manner [3]. This case highlights the need for more widespread recognition of this rare complication of Salmonella bacteraemia and more research into the clinical course of this condition.

## CASE REPORT

The patient was a 78-year-old Chinese gentleman who presented with fever, cough, diarrhoea and vomiting, of 1 day duration. Significant comorbidities included ischaemic heart disease (IHD), chronic obstructive pulmonary disease (COPD), bronchiectasis, peptic ulcer disease, hypertension and hyperlipidaemia.

Other than bilateral crepitations in the lung, the physical examination was otherwise unremarkable. Initial investigations were significant for a mild leucocytosis, acute kidney injury and mild hypokalaemia (Table 1). Chest and abdominal radiographs were unremarkable. Treatment was initiated for an infective exacerbation of bronchiectasis. Blood cultures and stool microbiology studies were sent off, and intravenous Co-amoxiclav and hydration were started.

**Table 1:** Summary of all relevant investigations results

Investigations	Results
White blood cell (3.37–8.38 × 10^9^/L)	12.80 × 10^9^/L
Absolute neutrophil (1.49–4.67 × 10^9^/L)	11.55 × 10^9^/L
Creatinine (59–104 umol/L)	118 umol/L
Total bilirubin (3–21 umol/L)	15 umol/L
Alanine aminotransferase (10–44 U/L)	14 U/L
Aspartate aminotransferase (10–34 U/L)	17 U/L
Alkaline phosphatase (45–122 U/L)	72 U/L
Gamma glutamyl transpeptidase (11–50 U/L)	17 U/L
Chest radiograph	Stable blunting of right costophrenic angle is likely related to pleural thickening. No confluent consolidation.
Abdominal radiograph	The visualized bowels are normal in esente and distribution. No gross bowel dilatation or air fluid levels seen. No definite radio-opaque urinary stone seen.
Ultrasound abdomen	No liver abscess. No biliary dilatation. Normal gallbladder. No hydronephrosis.
Computed tomography aortogram	There is no evidence of an aneurysm or mural flap to suggest the presence of a dissection. Appendix: Enlarged, fluid filled with hyperenhancement of the wall. It measures 11–12 mm in diameter with minimal adjacent fat stranding compatible with acute inflammation. Small esenteric nodes are seen in the right iliac fossa, likely reactive. No evidence of abscess or perforation. Findings suggestive of acute appendicitis.
Stool culture	Salmonella species
	Sensitive to Ampicillin, Ceftriaxone, Ciprofluoxacin, Cotrimoxazole
Stool for *Clostridium difficile*	Negative
Blood culture Day 1 of admission	Salmonella species
	Sensitive to Ampicillin, Ciprofloxacin, Ceftriaxone, Cotrimoxazole
Blood culture Day 10 of admission	No growth

**Figure 1:**
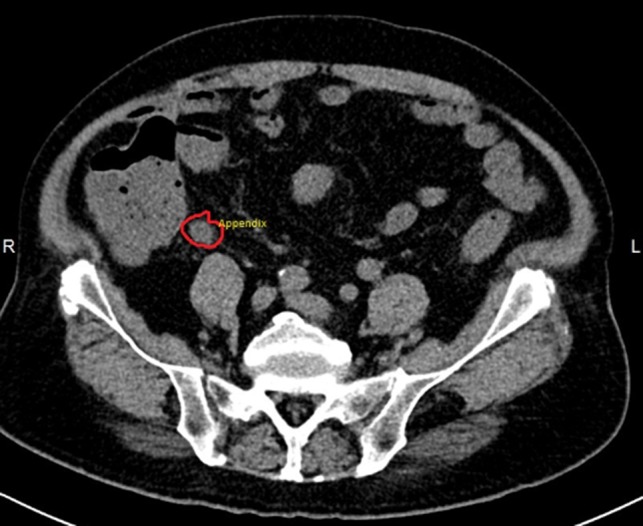
CT aortogram image showing the swollen appendix

Blood culture initially was reported as positive of gram negative rod. An ultrasound of abdomen was done. It showed no intra-abdominal abscess. Over the next few days, stool and blood cultures came back positive for non-typhoidal Salmonella species sensitive to ampicillin, ceftriaxone and cotrimoxazole. Salmonella serovar was not specified due to the hospital laboratory protocol. A computed tomography (CT) aortogram (Fig. 1) was done to look for aortitis as our patient has significant atherosclerosis. It did not show aortitis; however, an enlarged (11–12 mm in diameter), fluid filled appendix with hyperenhancement of the wall, minimal adjacent fat stranding and small mesenteric nodes in the right iliac fossa, was found. This was consistent with acute inflammation of the appendix and likely reactive lymphadenopathy. There was no evidence of abscess or perforation. A surgical and radiological consult was obtained, both agreed that the findings were in keeping with acute appendicitis. The decision was made to proceed with conservative management in view of poor premorbid status and multiple comorbid conditions.

The patient completed 2 weeks of intravenous ceftriaxone. Repeat blood culture did not show persistent salmonella bacteraemia. Acute kidney injury resolved and the patient was discharged well.

## DISCUSSION

Salmonella infections can be broadly classified into typhoidal and non-typhoidal salmonelloses [4]. Invasive systemic disease such as bacteraemia is a known clinical manifestation in typhoidal salmonella infections but uncommon in non-typhoidal salmonella infections which typically manifests as self-limiting gastroenteritis [4]. Appendicitis is a rare form of presentation of acute abdomen in Salmonella infections [5]. Van Noyen *et al.* [6] showed that only an estimated 8% of culture-proven bacterial enteritis had histologically proven appendicitis due to Salmonella. Two mechanisms on how Salmonella causes appendicitis have been described; either by direct invasion or via blood or lymphatics resulting in inflammation [3, 5].

This case report highlights the importance of recognizing appendicitis as a complication of enteric non-typhoidal Salmonella infection. Early recognition allows clinicians to facilitate appropriate management. Availability of microbiology result that confirms enteric bacterial infection would not or should not change the decision to treat appendicitis surgically [3]. In our case, the identification of appendicitis in our patient was an incidental finding on CT aortogram initially performed to rule out aortitis after detection of Salmonella bacteraemia. Although our patient was a poor candidate for surgical intervention, the positive cultures obtained from his stool and blood samples played an important role in his recovery by prompt use of culture-guided antibiotics. Steward-Parker *et al.* [5] described how timely microbiological analysis of stool and intraoperative pus samples were important for selection of the most appropriate antibiotics for eventual effective treatment. Some clinicians believe that intraoperative bacteriology in appendicitis is of minimal benefit [7], but several studies believe otherwise especially in the context of atypical presentations [7, 8]. Presentations such as diarrhoea associated with appendicitis, unresolved abdominal pain after appendicectomy and persistent signs and symptoms of infection should raise our index of suspicion for underlying Salmonella infection [7–10]. Several case reports have documented protracted course of recovery largely associated with late identification of Salmonella [3]. Culture results will facilitate early recognition of the enteric pathogen and guide antibiotic therapy based on sensitivities.

There is very little evidence on antibiotic responsiveness of Salmonella associated appendicitis [3]. Little is also known about their complication rates or clinical trajectory compared to other cases of appendicitis [3].

This is a rare case of acute appendicitis associated with culture-proven non-typhoidal salmonella infection. While less common as an infective source, it can be important to consider acute appendicitis in patients with Salmonella bacteraemia. More research needs to be done with regards to the clinical course of Salmonella related appendicitis.
